# Mitochondrial transplantation—a possible therapeutic for mitochondrial dysfunction? Mitochondrial transfer is a potential cure for many diseases but proof of efficacy and safety is still lacking

**DOI:** 10.15252/embr.202050964

**Published:** 2020-08-27

**Authors:** Robert N Lightowlers, Zofia MA Chrzanowska‐Lightowlers, Oliver M Russell

**Affiliations:** ^1^ Faculty of Medical Sciences Biosciences Institute Newcastle University Newcastle upon Tyne UK; ^2^ Faculty of Medical Sciences Translational and Clinical Research Institute Newcastle University Newcastle upon Tyne UK

**Keywords:** Membrane & Intracellular Transport, Molecular Biology of Disease, Regenerative Medicine

## Abstract

Transplantation of functional mitochondria directly into defective cells is a novel approach that has recently caught the attention of scientists and the general public alike. Could this be too good to be true?

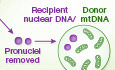

Mitochondrial dysfunction causes mitochondrial diseases, including various common neurodegenerative diseases, and is implicated in the ageing process. Consequently, it is now becoming a major target for biotechnology and pharma companies. To date, however, there have been few if any successes. The problems faced in fixing mitochondrial dysfunction range from identifying suitable targets to lack of methods that reliably transfect mitochondria. A novel approach has recently caught the attention of scientists and the general public alike: transplant fully functional mitochondria directly into defective cells. Could this be too good to be true?

## Mitochondrial disease

While many mitochondrial diseases affect neurological function, defective mitochondria can affect any tissue type and symptoms are often multisystemic and complex. To complicate matters further, the mitochondrion contains its own genome, mtDNA, that encodes just thirteen of the more than 1,000 polypeptides that comprise the mitochondrion. Pathogenic mutations that cause mitochondrial disease can thus occur in either genome. In general, adults tend to have mutations in mtDNA, whereas severe neonatal defects have aberrant nuclear genes. Taken together, mitochondrial disease is amongst the most common form of inherited neurological disorder with a minimum prevalence of 1:5,000.

## Current concepts for treating mitochondrial dysfunction

When treating pathogenic mtDNA defects specifically, it is important to remember that repairing all defective mitochondria is not necessary as the presence of a sufficiently large number of active mitochondria in a heterogeneous population can be enough to restore normal tissue function. Treatment strategies for mitochondrial dysfunction in general fall into the following categories: (i) increasing mitochondrial biogenesis; (ii) reducing dysfunctional mitochondria and replacing them with active ones; (iii) by‐passing or substituting the defective component; (iv) targeting the consequences of mitochondrial dysfunction; or (v) reprogramming metabolism (Russell *et al*, 2020). Certainly, identifying methods to promote mitochondrial proliferation is an appealing strategy. For example, careful exercise still is one of the most promising therapeutics for mitochondrial disease, although whether it increases mitochondrial biogenesis as might be expected is unclear. Proliferation as a method for rescuing mitochondrial function is also supported by experiments in mice with mitochondrial disease that overexpressed PGC1alpha, a key mediator of mitochondrial biogenesis. It shows that, least in some cases of mitochondrial dysfunction, it is beneficial to have a larger number of mitochondria *in toto* even if a proportion is dysfunctional. This should be balanced with the observation that rapamycin treatment of another respiratory‐deficient mouse model helped delay symptoms, possibly through promoting increased turnover of dysfunctional mitochondria.

One concept that was not addressed in the above‐mentioned review (Russell *et al*, 2020) is whether increasing mitochondrial bulk by mitochondrial transplantation *per se* could be a viable approach—which we address in this article.

## Increasing mitochondrial mass by transplanting “normal” mitochondria into affected cells

**Figure 1:**
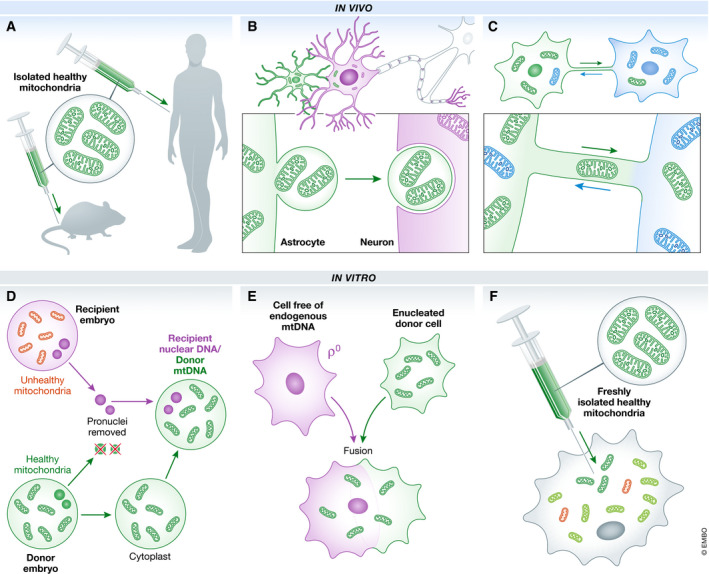
Ways in which mitochondria could potentially be transferred between cells Isolated wild‐type mitochondria have been directly injected into rodent models or patients (A). Reports have suggested that at sites of cerebral ischaemia, mitochondria can be encapsulated as a vesicle for transfer from astrocytes and endocytosed by neurons (B). Another possible method of *in vivo* trafficking of mitochondria between cells is through fine open‐ended extensions termed nanotubes (C). A licensed method is pronuclear transfer whereby cytoplasts harbouring healthy mitochondria from a healthy embryo are combined with the pronuclei of another embryo (D). Combining mitochondria with the content of enucleated cells that lack mitochondria is a technique that has been extensively used to generate cybrid cell lines (E). Isolated healthy mitochondria could be injected into cells harbouring both healthy (blue) and dysfunctional (red) organelles (F).

Surprisingly, there are many reports which claim that such transplantation in cells, tissue, model systems and even in patients, either autologous or non‐autologous, has been effective. These include myocardial ischaemia/reperfusion injury, spinal cord injury and models of Parkinson's disease where, remarkably, efficacy has been reported merely after intravenous introduction of mitochondria (Fig 1, panel A).

Mitochondrial transfer has even been observed naturally *in vivo*: in response to a focally induced cerebral ischaemia, astrocytes close to the site of lesion transferred mitochondrially incorporated particles by an endocytotically mediated CD34^+^ controlled process into neurons (panel B), (Hayakawa *et al*, 2016). Interestingly, the authors were careful to point out that quantitative thresholds to produce any functional benefit had not been assessed. Tunnelling nanotubes (TNTs) have also been implicated as mediators of intercellular mitochondrial transport. These cytoskeletal‐based open‐ended tubular extensions enable direct exchange of metabolites, proteins, viral particles and organelles between cells in culture and *in vivo*, often as a consequence of stress (panel C). Indeed, mitochondrial transfer via TNT has been observed between pluripotent stem cells and a variety of recipient cell types *in vitro*, often leading to increased ATP production, oxygen consumption and cell viability (reviewed in ref. Murray & Krasnodembskaya, 2019). In addition, extracellular vesicles, essentially membrane‐bound cellular secretions, can occasionally contain mitochondria. Islam *et al* (2012) reported that mitochondria were transferred by mesenchymal stem cells to lung alveolar epithelial cells and observed a consequential increase in ATP levels in the recipient cells. The epithelial cells were shown to produce both microvesicles and TNTs to promote mitochondrial transfer.

Certainly, all these data challenge our understanding of how organelles behave in cells, and we should consequently demand high levels of rigour to support these claims. This has become even more essential as clinical trials using mitochondrial transplantation are being planned and performed. However, if the processes described in these reports are robust, it would have great potential for mitochondrial therapeutics.

## Do mitochondria injected or imported into cells retain activity?

Work in many laboratories has showed that enucleated cytoplasts can be reconstructed with donor nuclei, which has led to the concept of mitochondrial replacement therapy to prevent the transmission of defective mitochondria between generations (Fig 1, panel D). This method should not be confused with attempts to supplement oocytes with either small amounts of ooplasm or directly with mitochondria isolated from adult oogonial stem cells to increase fertility rates in IVF. Both methods raised substantial concerns regarding efficacy and safety (reviewed in (Kristensen *et al*, 2017). In many well‐documented cases, cytoplasts have also been used to transmit functional mitochondria to cells devoid of mtDNA, producing transmitochondrial cybrids (panel E).

In all these cases, mitochondria were not isolated but transferred along with cytosol. Numerous control experiments confirm that restoration of function is critically dependent on transferring *active* mitochondria. Thus, the question whether isolated mitochondria retain activity when they are injected directly into cells or tissues is particularly important as isolated mitochondria are now being used to treat patients, either with mitochondrial disease or myocardial ischaemia/reperfusion injury.

With respect to whether mitochondria fully retain their integrity outside the cytoplasm, we have known for many years that this is possible for isolated organelles but only when prepared carefully in specifically supplemented media. This is a key issue, because the retention of a robust membrane potential is essential for normal function. For example, it is extremely challenging to revive mammalian mitochondria that have been stored below 4°C, as ice crystals destroy their integrity. Amongst many other stresses, injection of isolated mitochondria into an extracellular milieu would exert a substantial change in calcium ion concentration. This could cause rapid and potentially irreversible loss of the mitochondrial membrane potential owing to the opening of the mitochondrial permeability transition pore. Maintenance of a high membrane potential is arguably the most important function of mitochondria and is equally crucial when injecting them directly into cells, where they would be expected to fuse with a functional mitochondrial network. The inference, stretching back to experiments in the 1980s, suggests that it may be possible—at least for careful injection of freshly isolated mitochondria (panel F)—but the efficiency of this process is extremely poor, and without a strong selection for the injected mtDNA and time to facilitate the selection, the overall effect on cell function will be minimal. Irrespective, mitochondria, particularly those that have lost their membrane potential, are constantly turned over in the cell inferring that if transplanted mitochondria had any benefit, this may be just relatively short‐lived.

Considering all these caveats, it is difficult to see how transplantation could lead to long‐term benefits directly from the transplanted mitochondria. Could some of these remarkable claims be explained by more indirect effects? Various reports on mitochondrial transplantation focus on ameliorating the effects of reperfusion injury following ischaemia in cardiac tissue. These have led to pilot trials with a small number of paediatric patients with cardiac ischaemia who were undergoing ECMO (extracorporeal membrane oxygenation); a similar trial is currently recruiting (NCT02851758). Positive effects were reported with 4 of 5 patients showing improved ventricular function (Emani *et al*, 2017) but these studies have been questioned (Bertero *et al*, 2018). The group conducting these studies has previously suggested that at least part of the beneficial results could be caused by extracellular effects. Perhaps extracellular mitochondria could promote a signalling cascade merely by being in proximity to struggling cells? This hypothesis would have the double advantage as it may explain how such a vanishingly low amount of imported organelle could have such a strong protective effect. Such positive effects have also been seen in other mitochondrial transplant experiments and could similarly explain the kinetics of protection, which appears to happen before the organelles enter the cell. However, as control experiments have shown that the protective effect requires functioning mitochondria, it still does not explain how injected purified mitochondria could retain their activity during the isolation and transfer process.

In an ischaemic pig heart model, prelabelled mitochondria were found up to 4 weeks after injection, although the authors did not analyse their functionality (Kaza *et al*, 2017). More recent bioenergetic studies on isolated mitochondria transferred into cardiomyocytes reported beneficial consequences on OXPHOS 2 days postinjection (Ali Pour *et al*, 2020), but this positive effect was not maintained. The same study noted a similar benefit with non‐autologous and even with interspecies transfer. The latter is particularly surprising, as the mitochondrial genome cannot naturally be expressed in such interspecies crosses. This would imply that, for interspecies transfer to produce a functional effect after 2 days, the imported mitochondrial protein would have to be retained, functional and not degraded during this period.

The preservation of an enduring effect will depend on the active expression of wild‐type mtDNA. Thus, a likely explanation for any positive effect resulting from mitochondrial transfer is that the transplanted mitochondrion is merely a vector for delivering normal mtDNA to the mitochondrial network. This would potentially be highly advantageous, as there is currently no non‐invasive method for delivering DNA to the organelle. Increased copy number of wild‐type mtDNA may be very useful, particularly since many mtDNA mutations are functionally recessive. Irrespective, it would be surprising if overall copy number did not revert to normal levels, and the remarkably rapid effects reported about transfer of some mitochondria would not be fully compatible with this hypothesis.

If mitochondrial transplantation can effectively deliver normal copies of mtDNA to the mitochondrial network, and in a way that permits it to be expressed naturally, how could the process be used to treat patients? One possible approach is to transfer mitochondria to stem cells *ex vivo*. Intriguingly, a previous study showed that muscle stem cells, so‐called satellite cells, when activated by induced muscle degeneration, were able to repair mitochondrial respiratory deficiency in the muscle of a patient with a mitochondrial disorder who carried both normal (low levels) and pathogenic (high levels) forms of mtDNA. In this case, the wild‐type mtDNA was supplied by the endogenous activated satellite cells, but, unfortunately, rescue was only possible in a very small area of muscle. To be of use therapeutically, it must somehow increase the levels of normal mitochondria in a large number of tissue stem cells. This is currently being tested in a clinical trial for patients with a specific mtDNA disease, Pearson's syndrome by injecting unaffected mitochondria into patient stem cells *ex vivo*.

## The Pearson's trial

Pearson bone marrow syndrome is a rare and severe mitochondrial disease affecting neonates, caused by deletions in the mitochondrial genome. Like in so many instances of mitochondrial disease, tissue specificity is profound and can vary between patients but the rationale behind this specificity is currently unknown. Amongst many other symptoms, the bone marrow and pancreas are particularly affected, resulting in pancytopoenia (a major defect in the size and number of all types of blood cells), severe stomach pain and diabetes. There is no cure, and patients eventually die of profound lactic acidaemia, sepsis or liver failure. Three children with Pearson's syndrome and one with the related Kearns–Sayre syndrome were already subjected to a mitochondrial augmentation therapy under a compassionate care programme, which was the foundation for a phase I/II clinical trial (NCT03384420) that started in June 2019. CD34^+^ haematopoetic and stem cell progenitors were harvested from the children and subjected to enrichment with isolated maternal mitochondria *ex vivo* before intravenous infusion. Unfortunately, the resultant data had not been peer reviewed when it was presented at the American Society for Hematology meeting in December 2018; a summary is provided on the Minovia website. Remarkably, aerobic capacity and ATP content were reported to increase in lymphocytes of two patients, leading to improved quality of life. The subsequent phase I/II clinical trial is currently underway, with the aim of further assessing the safety and efficacy of mitochondrial augmentation.

## Concluding remarks

It is becoming increasingly apparent that mitochondrial dysfunction is associated with many different clinical defects. As there is currently no reliable therapeutic that can rescue mitochondrial function, we should welcome all advances that are grounded in thorough research. Numerous reports indicate that defects can apparently be ameliorated by either endogenous or exogenous mitochondrial transplantation. We should consider these approaches with an open mind, providing the supporting data have been thoroughly validated. For patients with a poor prognosis, such as those children with Pearson's syndrome, it is argued that any treatment that shows any potential of success could be considered even if we may not understand exactly how the procedure works. The key to progress is that we must be convinced that the treatment is indeed working. It will certainly be interesting to follow the outcome of this trial.
